# Controlling intercellular flow through mechanosensitive plasmodesmata nanopores

**DOI:** 10.1038/s41467-019-11201-0

**Published:** 2019-08-08

**Authors:** Keunhwan Park, Jan Knoblauch, Karl Oparka, Kaare H. Jensen

**Affiliations:** 1 Department of Physics, Technical University of Denmark, DK-2800 Kgs. Lyngby, Denmark; 2 Institute of Molecular Plant Sciences, University of Edinburgh, Edinburgh, EH9 3BF UK

**Keywords:** Membrane biophysics, Applied mathematics, Plant development, Plant cell biology

## Abstract

In plants, plasmodesmata (PD) are nanopores that serve as channels for molecular cell-to-cell transport. Precise control of PD permeability is essential to regulate processes such as growth and tissue patterning, photoassimilate distribution and defense against pathogens. Callose deposition modulates PD transport but little is known of the rapid events that lead to PD closure in response to tissue damage or osmotic shock. We propose a mechanism of PD closure as a result of mechanosensing. Pressure forces acting on the dumbbell-shaped ER-desmotubule complex cause it to be displaced from its equilibrium position, thus closing the PD aperture. The filamentous protein tethers that link the plasma membrane to the ER-desmotubule complex play a key role in determining the selectivity of the PD pore. This model of PD control compares favorably with experimental data on the pressure-generated closure of PD.

## Abstract (web summary)

Plasmodesmata channels connect neighbouring plant cells and respond to external stimuli via changes in permeability. Here Park et al. propose that mechanical forces can displace the dumbbell-shaped ER-desmotubule complex that spans the central plasmodesmatal cylinder leading to closure of the pore.

## Introduction

Living organisms contain distinct sub-compartments to facilitate regulation of physiochemical processes and biological functions. In addition to organs and tissues, both cells and subcellular organelles are physically separate from their surroundings, yet remain coherent with their neighbors and able to exchange specific sets of molecules. The channels linking distinct domains in living organisms play essential roles in biological processes across multiple scales; for instance in growth and tissue patterning^1,2^, nutrient and energy distribution^3^, and defense against pathogens^4^.

Plasmodesmata (PD) are intercellular nanochannels in plants that facilitate transport of small molecules such as ions, hormones, and photosynthates. PD pores traverse the cell wall and directly link the cytoplasm of neighboring cells. Intercellular transport occurs by a combination of diffusion and advection^5^. PD in higher plants have the capacity to dynamically regulate their permeability to facilitate trafficking of macromolecules such as transcription factors and RNAs, and to defend against pathogen invasion^6^. Permeability can change during development and in response to environmental signals by deposition of the carbohydrate callose at the PD entrance^7^.

However, PD pores can also respond rapidly to cellular damage and osmotic shock. For instance, a difference in cell turgor of 200 kPa instantly reduces PD transport by ~50% between adjoining trichome cells of *Nicotiana clevelandii*^8^, and turgor differences that arise during cell growth have also been associated with reduced permeability^9–12^. The rapid reduction in transport cannot be explained by standard models of PD transport, which assume that cell–cell movement occurs by a combination of molecular diffusion and bulk flow in static PD geometries^13–15^. The physical mechanism of pressure regulation of the permeability remains unknown.

**Fig. 1:**
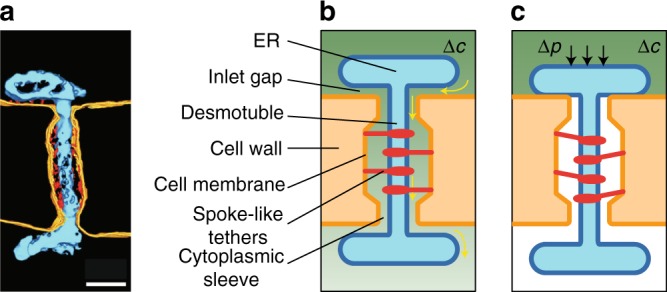
Mechanosensitive plasma membrane (PM) nanopores. **a** TEM tomography of a PD highlighting the cell membrane (yellow lines), ER-desmotubule complex (blue) and spoke-like tether protein filaments (red dots). Scale bar, 50 nm. **b** Schematic highlighting significant structural components and transport processes. Solute transport (yellow arrows) across the PD is driven by a concentration gradient Δ*c* (high concentration = dark green, low concentration = light green). **c** Mechanical effects on PD permeability: An intercellular turgor pressure difference Δ*p* leads to a displacement of the ER-desmotubule complex towards the cell wall, thus reducing the pore permeability. See details in the text. Panel **a** adapted from ref. ^26^, reproduced with permission

The pore structure, however, may hold clues to the origin of this effect. PD are cylindrical nanopores, typically 300 nm long and 30 nm wide, that cross the wall between plant cells. The pores are open, that is the plasma membrane (PM) of adjacent cells meet inside the pore. The cortical endoplasmic reticulum (ER) permeates each PD, and the gap between the cylindrical desmotubule and the PM forms an annular cytoplasmic sleeve though which water and solutes move (Fig. 1). The ER-desmotubule complex is anchored by filamentous protein tethers. A pressure difference Δ*p* between neighboring cells will displace the ER-desmotubule complex from its equilibrium position but this motion is resisted by the spoke-like tethers. The change in pore geometry will modify the aperture of the inlet gap, and hence the pore permeability.

Accordingly, we raise in this report the question of mechanical effects on PD permeability. Specifically, we examine the consequence of stress-induced changes on the equilibrium position of the desmotubule complex, and the resultant effects on diffusive and advective transport. As we demonstrate below, the pores indeed display a predicted pressure-dependent permeability that fits closely with experimental data. Strikingly, material parameters measured in PD are consistent with our predicted magnitude of the closing pressure. In a broader biomimetic perspective, our findings point to general design rules for flow control in artificial nanopores.

## Results

### Mechanosensitive control of PD permeability

The ability to sense mechanical cues is a well-established component of cells in all branches of life^16^. Mechanosensitive (MS) ion channels translate mechanical forces generated within cells and by interactions with neighboring cells into a trans-membrane ion flux that triggers the desired stimulus response. In plants, MS-channels are known to be involved in several essential processes, including membrane tension regulation, movement, and protection from osmotic shock^17,18^. The presence of rigid cell walls, however, limits membrane stress and direct contact between adjacent cell membranes is rare. To facilitate cell-to-cell communication, plants (and some algae) use PD nanopores to transfer solutes, metabolites, and macromolecules between cells. There is experimental evidence to suggest that PD permeability is modified in response to mechanical stress, yet the detailed mechanism is unknown^8^.

The question of PD permeability dates back to the early history of plant science. In 1879, Edouard Tangl observed fine linear marks in the walls between certain plant cells and described them as ‘conducting ducts’^19^. His discovery led to the radical ‘symplast’ concept, which describes the unique PD-mediated continuity between plant cells. It has since emerged that PD allow for continual cell-to-cell communication in numerous integral processes such as tissue patterning, photoassimilate distribution, defense signaling, and the spreading of viruses.^1–3,9–12,20–25^. In all cases, accurate temporal and spatial control of PD permeability is essential.

**Fig. 2:**
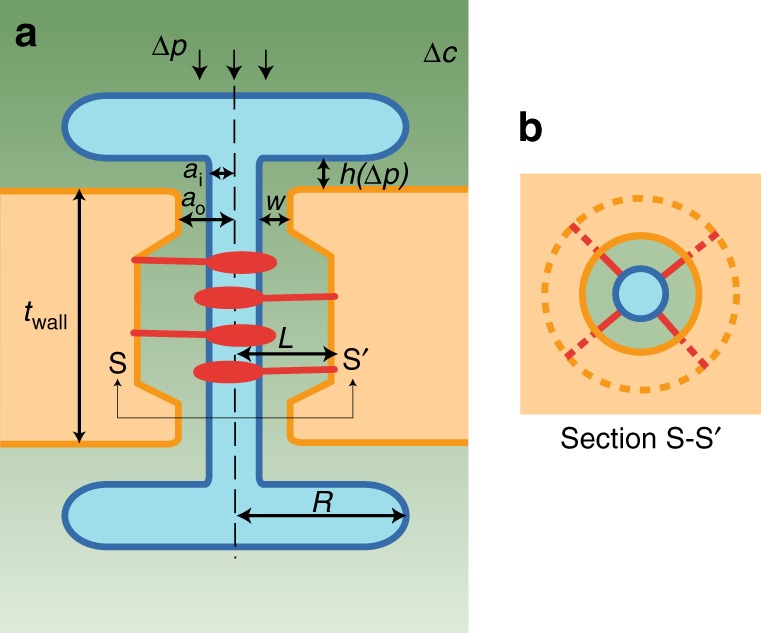
Schematic of PD pore geometry. **a** The structure is characterized by the cell wall thickness *t*_wall_, pressure-dependent inlet gap height *h*(Δ*p*), inlet gap radius *R*, desmotubule radius *a*_i_, PD pore radius *a*_o_, and the cytoplasmic sleeve thickness *w*. **b** Section view of the PD, highlighting the annular pore structure. See legend in Fig. 1 and details in the text

While current theories are able to describe transport in static PD geometries^13–15^, this conceptual framework is insufficient to account for potential pressure-induced changes in PD permeability (Fig. 1c). Hence, we must include the effects of mechanosensitivity on the pore structure. Again, the pore geometry may hold clues to the mechanism with which pressure impacts permeability. In the event of an intercellular pressure difference, the mechanical forces acting on the dumbbell-shaped ER-desmotubule complex will cause it to be displaced from its equilibrium position. As shown in Fig. 1 and sketched in Fig. 2, a number of spoke-like tethers link the desmotubule to the PM along the cytoplasmic sleeve. These comprise filamentous proteins that provide a strong bond and will resist the pressure-induced movement^26^.

The spoke-like tethers have been widely observed in a broad range of land plants and appear to be ubiquitous among dicots. They were described by Ding et al.^27^, Badelt et al.^28^, and more recently in great detail by Nicolas et al.^26^. They seem to be absent in newly formed PD but form during subsequent development, and the majority of models of PD architecture include the tethers/spokes^26,29–31^. We restrict our analysis to mature PD in which tethers appear to be omnipresent. The application of a pressure gradient across the cell wall can also affect other cellular properties. For instance, the structure of the protein tethers could be influenced by the application of pressure. However, the forces considered here (Δ*p* ~ 1 bar) are significantly smaller than those required to achieve significant conformation of proteins^32^. Similarly, the magnitude of the elastic deformation of the cell wall is of the same order as the thickness *t*_wall_, hence the bending-induced changes to the pore aperture are relatively small. Finally, we note that and the presence of callose could mitigate the effects of stress concentration, a process in which a geometric discontinuity (the PD pore) leads to a several-fold amplification of forces and thus the possibility of fracture and subsequent crack formation^33–36^.

To determine the equilibrium position of the ER, we express the coupling between the applied load and the elastic response from the tethers as a force balance *F*_p_ + *F*_e_ = 0. Here, $F_{\mathrm{p}}\sim \Delta p\pi a_{\mathrm{o}}^2$ is the pressure force acting on the effective top ER surface area $\pi a_{\mathrm{o}}^2$, and *F*_e_ ~ *k*Δ*h* is the elastic force generated in response to a displacement Δ*h* of the tethers along the vertical *z*-axis with an effective spring constant *k* (Fig. 2). We assume that the deflection of the tethers follows the bending of a cantilever beam fixed at the PM and subjected to a point load at the desmotubule. Thus, the effective spring constant^37^ can be written as *k* = 3*nEI*_tether_*L*^−3^, where *n* ~ 100 is the number of tethers, *E* ~ 2 GPa is Young’s modulus, *I*_tether_ = *πd*^4^(64)^−1^ is the area moment of inertia, and *d* = 1 nm and *L* = 9 nm is the filament diameter and length, respectively^26,38^.

This leads to an effective spring constant *k* = 0.0404 N/m and the displacement from vertical equilibrium can be written as

1 $$h(\Delta p) = h_0\left( {1 - \frac{{\pi a_{\mathrm{o}}^2}}{{h_0k}}\Delta p} \right),$$

where *h*_0_ is the equilibrium gap size. Accordingly, the inlet aperture will decrease in size by $\pi a_{\mathrm{o}}^2k^{ - 1}\sim 0.82$ nm for every 100 kPa difference in cell turgor. For an equilibrium gap is *h*_0_ = 2.8 nm, the PD is completely blocked at the critical closing pressure $\Delta p_c = h_0k(\pi a_{\mathrm{o}}^2)^{ - 1}\sim 340$ kPa.

Having established the variation in gap aperture with the applied pressure, we are now in a position to quantify the pressure-dependent PD transport properfies. Trafficking of small molecules across PDs occurs primarily by molecular diffusion. Other processes, such as electrophoresis and bulk flow, also potentially contribute but we do not consider these effects here. Specifically, we assume a steady-state system in which the solute is electrically neutral and the Peclet number *Pe* = *ut*_wall_*D*^−1^ is small, where *u* is the characteristic bulk flow speed and *D* is the diffusion coefficient.

With these assumptions, the magnitude of the molecular current *I* follows from solutions to the steady-state diffusion equation *D*∇^2^*c* = 0. The link between *I* and the concentration difference can be expressed as (see “Methods”)

2 $$I = \frac{{\Delta c}}{{R_{\mathrm{d}}}} = P\Delta c,$$

where we have introduced the pressure-dependent diffusive resistance *R*_d_^39^ and permeability *P* = (*R*_d_)^−1^ given by

3 $$R_{\mathrm{d}}(\Delta p) = \frac{{t_{{\mathrm{wall}}}}}{{DA_1}}\left[ {1 + \frac{{A_1}}{{t_{{\mathrm{wall}}}}}\frac{{\ln (R/a_{\mathrm{o}})}}{{2\pi h(\Delta p)}}\frac{1}{{H(\lambda )}}} \right].$$

The first term in the bracket corresponds to the cytoplasmic sleeve, and the second term is the inlet gap resistance. In Eq. (3), *t*_wall_ = 300 nm is the pore length, *D* = 2 × 10^−10^ m^2^ s^−1^ is the diffusion coefficient of fluorescent dye carboxyfluorescein, $A_1 = \pi (a_{\mathrm{o}}^2 - a_{\mathrm{i}}^2)$ is the conductive area of the cytoplasmic sleeve with inner radius *a*_i_ = 7.5 nm and outer radius *a*_o_ = 10.3 nm and *R* is the outer radius of the ER-complex^3,40–42^. We estimate that *R* = 2*a*_o_ (Fig. 2 and ref. ^3^), and assume in the model that the outer radius *a*_o_ is independent of *z* and thus constant throughout the pore, which provides a conservative estimate of the resistance. In summary, the pressure-dependent parameters in Eq. (3) are the gap height *h*(Δ*p*), see Eq. (1), and the diffusion hindrance factor *H(λ*) which depends only on the solute-to-pore size ratio *λ* = *s*(*h*(Δ*p*))^−1^, where *s* is the effective diameter of the solute (see “Methods”, Eq. (13)).

**Fig. 3:**
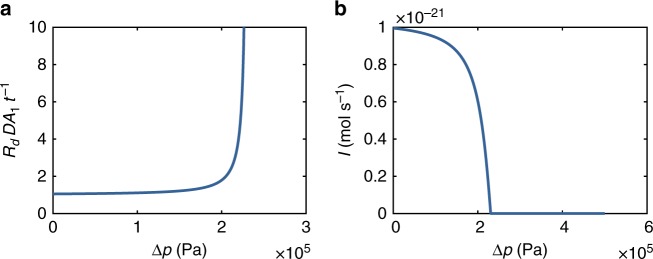
The impact of pressure on PD resistance and transport. **a** Normalized diffusion resistance *R*_*d*_*DA*_1_*t*^−1^ (Eq. (3)) plotted as a function of turgor pressure difference Δ*p*. **b** Diffusion current *I* = Δ*c*(*R*_*d*_)^−1^ plotted as a function of turgor pressure difference Δ*p* for a concentration difference Δ*c* = 10 μM. The transport currents decline in sync with increasing resistance

The behavior of the mechano-sensitive diffusion resistance (Eq. (16)) as function of intercellular pressure difference Δ*p* can be divided into two distinct regimes (Fig. 3). At low pressures, when the inlet gap is slightly deformed, the resistance increases slowly and transport through the cytoplasmic sleeve is the limiting process. By contrast, at relatively high pressures, the combined effects of the reduced transport area in the inlet gap and steric effects lead to a sudden and dramatic increase in resistance and consequent reduction in transport.

### Effects of pore deformation on transport properties

**Fig. 4:**
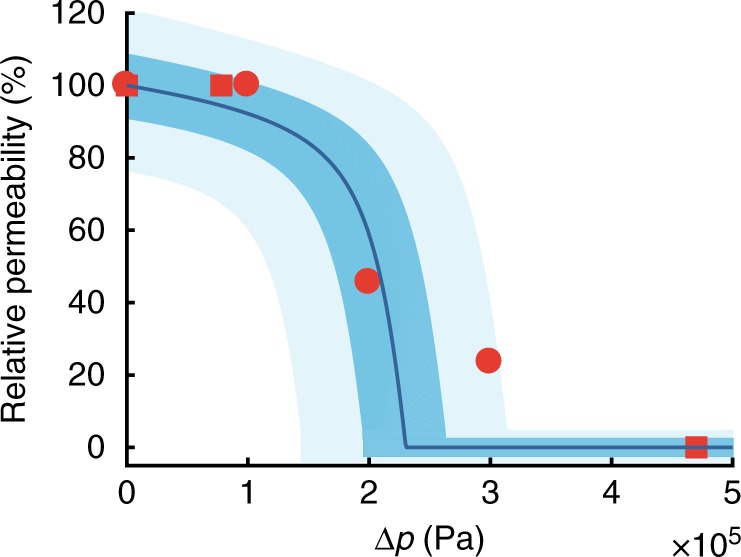
Impact of pressure on PD permeability. Relative permeability *P*(*P*_0_)^−1^ is plotted as a function of applied pressure Δ*p* (solid line). The two shaded regions indicated the sensitivity to the cytoplasmic sleeve width *w* = *a*_o_ − *a*_i_ (±10%, and ±25%). The PD’s ability to mediate transfer of solutes decline in sync with increasing pressure. Experimental data (dots and squares, from ref. ^8^ and ref. ^9^, respectively) compare favorably with the model. Source data are provided as a Source data file

The preceding analysis of transport in a mechanosensitive PD revealed a strong impact of turgor gradients on diffusive pore permeability (Fig. 4). The permeability decreases slowly at low pressures when the inlet gap is only slightly deformed. Around Δ*p* ~ 150 kPa, however, permeability dramatically decreases and the inlet gap is effectively blocked at Δ*p* = 225 kPa. This occurs earlier than the pressure required for closure (Δ*p*_*c*_ ~ 340 kPa (see Eq. (1)), because steric effects reduces the effective diffusivity in the pore before it is completely occluded. To test these predictions, we compare our synthesis with existing data. Oparka and Prior^8^ generated pressure differentials between adjacent leaf trichome cells of *Nicotiana clevelandii* using a modified micropressure probe/injection system. Intercellular transport of a fluorescent tracer was monitored to quantify the impact on PD permeability. The experiments showed a gradual decrease in permeability with increasing pressure, and that elevations of cell turgor in excess of 200 kPa were required to strongly impede intercellular transport. The structure of Nicotiana PD was studied in detail by Faulkner et al.^43^ using cryofracturing. Their data showed that trichome plasmodemata display the characteristic development of PD, including cytoplasmic sleeves and dimensions similar to other species. Similarly, in experiments on cotton fiber elongation, Ruan et al.^9^ observed no impact on permeability when the turgor difference was Δ*p* ≤ 80 kPa, but reported complete closure at Δ*p* = 470 kPa.

The qualitative trends and quantitative behavior of these data agree with our model (Fig. 4). Differences, however, are apparent and we attribute these to variations in the pore geometry not capture by our model. The two shaded regions in Fig. 4 illustrate the dependence on the slit width *w*, and we note that majority of the data can be rationalized by a 10%-variation in *w*.

### Impact of osmotic interactions on PD permeability

**Fig. 5:**
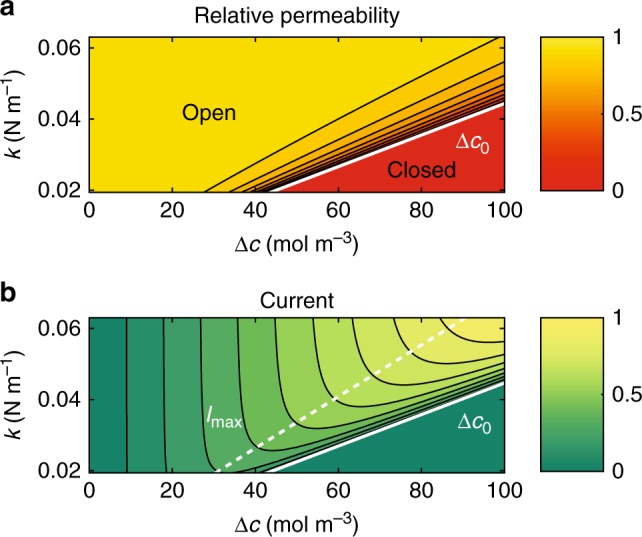
The impact of osmotic effects on PD transport. **a** Contour plot of the relative permeability *P* as a function of osmotic concentration difference Δ*c* and tether flexibility *k*. The values are normalized by the permeability of the fully open state *P*(Δ*p* = 0). The permeability remains constant (yellow) until it abruptly decreases and the pore closes at the critical concentration Δ*c*_0_ (red), that increases with *k* (thick diagonal white line). **b** Contour plot of the PD current *I* as a function of the osmotic concentration difference Δ*c* and tether flexibility *k*. The values are normalized by the maximum current (light green) obtained for the range of parameters shown. The current increases in proportion to the product of permeability *p* and concentration Δ*c*. For constant *k*, the current it reaches a peak value *I*_max_ as concentration approaches Δ*c*_0_ (dashed line)

Osmosis is a key process in cell expansion^44^, and osmotic stress, caused by e.g., drought or salinity factors, is an important physical process with adverse effects on cell growth and plant productivity^45,46^. Differences in osmotic pressure between neighboring cells can lead to a PD-mediated liquid current that contributes to lysis or plasmolysis, or a reduction in growth rate. In the preceding analysis of PD transport regulation, we considered independent pressure and concentration gradients. However, in the case of osmotic processes, the two are intrinsically linked. To elucidate the impact PD mechanosensitivity on strong osmotic effects, we consider the case when the presence of a solute imbalance of concentration Δ*c* induces an osmotic pressure difference between the two cells of Δ*p* = *RT*Δ*c*. The osmotic pressure difference Π(Δ*c*) generated by a concentration imbalance Δ*c* between two cells is a non-linear function of Δ*c* and the membrane transport coefficients. Here we use the van’t Hoff value for the osmotic pressure Π(Δ*c*) = *RT*Δ*c*, which is valid only for dilute solutions and ideal membranes. At moderate concentrations of small molecules (e.g., *c* < 1 M for NaCl and *c* < 0.5 M for sucrose), the error in the osmotic pressure introduced by using the van’t Hoff value is ~10%^47^. Specifically, we solve Eqs. (2) and (3) where the diffusion resistance is now a function of the concentration difference Δ*c*, such that *R*_d_ = *R*_d_(Δ*p* = *RT*Δ*c*). As shown in Fig. 5, PD transport proceeds unimpeded at low-to-moderate concentration gradients. By contrast, PD permeability is strongly reduced at higher concentrations, and the threshold is a function of the effective ER-desmotubule complex flexibility (parameter *k*, see Eq. (1)). Below this threshold, established tissue-scale models of cell–cell transport remain valid (e.g., ref. ^48^). However, during strong osmotic stress, mechanosensitive PD could provide effective protection against symplastic water loss which is not captured by established models.

## Discussion

PD are enigmatic structures of paramount importance to numerous processes yet relatively little is known of their role in mechanosensing. In this paper, we have described a model of pressure-regulated PD permeability in which pressure-induced movement of the ER-desmotubule complex strongly impacts PD transport.

The analysis suggests a number of specific experimental investigations. Chief among them are detailed studies of PD permeability, using appropriate tracer particles, to examine the response to turgor gradients and their importance in regulating intercellular transport. However, conventional techniques measure pressure and introduce fluorescent tracer molecules using mcirocapillary probes, and overcoming the stress introduced by such invasive techniques remains a challenge^49,50^. Moreover, symplastic dyes are not available in all sizes and thus cannot provide a complete picture of the size exclusion limit, nor can they characterize PD-protein interactions that selectively modify permeability.

Nevertheless, the predicted variations in transport rate across the cell surface suggest the possibility that the wound response might occur autonomously, and that cells subjected to osmotic shock above a certain critical level could be symplastically isolated from their neighbors. To fully characterize these processe, it would be important to develop methods able to separate out the (relatively slow) impact of callose deposition from rapid mechano-sensitive PD responses. Moreover, permeability in synthetic systems that involve nanopores could be controlled by similar processes, e.g., using vesicles trapped in small pores^51^. Finally, the connection between the proposed mode of PD mechanosensing and the transport of pathogens remains to be elucidated.

Taken together with recent work on the ultrastructure of PD pores^26^, and the multitude of processes in which they are involved^6^, the present analysis serves to highlight the unusual features of mechanosensitivity in plants.

## Methods

### PD transport by molecular diffusion

**Fig. 6:**
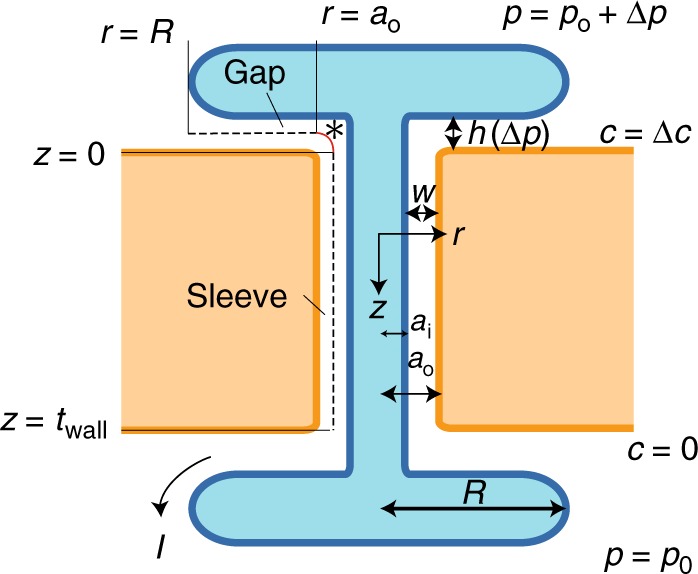
Schematic of the PD pore geometry. Transport of molecules by molecular diffusion is driven by a difference in solute concentration Δ*c* across the cell wall. The diffusive current *I* is determined from solutions in the gap and sleeve domains (see Eqs. (9) and (10)). The short gap-sleeve transition region of length *w*~*h* (indicated by an asterisk) is not included in the analysis. See also Figs. 1 and 2

In this section, we consider transport by molecular diffusion in a PD pore. Assuming steady-state conditions, the governing equation is the diffusion equation

4 $$D\nabla ^2c = 0,$$

subject to boundary conditions of constant concentration (and pressure) on either side of the cell wall (Fig. 6)

5 $$c = \Delta c\quad {\mathrm{and}}\quad p = p_0 + \Delta p\quad {\mathrm{upper}}\,{\mathrm{cell,}}$$

6 $$c = 0\quad {\mathrm{and}}\quad p = p_0\quad {\mathrm{lower}}\,{\mathrm{cell}}{\mathrm{.}}$$

Moreover, we assume that the molecular flux ***j*** = −*D***∇***c* vanishes on all solid boundaries. To characterize the transport properties of the PD pore, we use solutions of Eqs. (4)–(6) to compute the total diffusive current $I = {\int} {\boldsymbol{j}} \cdot {\boldsymbol{n}}{\mkern 1mu} {\mathrm{d}}A$.

In the following, we use that the PD has two distinct geometric features: the inlet gap and the cytoplasmic sleeve (Fig. 6). Transport in both elements is one-dimensional because the geometries are shallow ($h_0/R \ll 1$ and $w/t_{{\mathrm{wall}}} \ll 1$). In the gap we therefore assume that the concentration can be written as *c* = *c*_*g*_(*r*), where *a*_o_ < *r* < *R*, while in the sleeve *c* = *c*_*s*_(*z*) where 0 < *z* < *t*_wall_. The two elements meet in a region of length *w* ≈ *h* at the gap-sleeve interface. However, because this domain is much shorter than both the sleeve and gap ($w \ll R$ and $w \ll t_{{\mathrm{wall}}}$) the impact of this region on the transport process is negligible. The governing equations and boundary conditions are thus

7 $$\frac{1}{r}\frac{\partial }{{\partial r}}\left( {r\frac{{\partial c_g}}{{\partial r}}} \right) = 0,\qquad c_g(R) = \Delta c,\quad c_g(a_{\mathrm{o}}) = c^ \ast$$

8 $$\frac{{\partial ^2c_s}}{{\partial z^2}} = 0,\qquad c_s(t_{{\mathrm{wall}}}) = c^ \ast ,\quad c_s(0) = 0$$

where c^*^ is an unknown intermediary concentration at the interface. The solutions are:

9 $$c_g = \left( {\Delta c - c^ \ast } \right)\frac{{\log (r/a_{\mathrm{o}})}}{{\log (R/a_0)}} + c^ \ast ,$$

10 $$c_s = \frac{z}{{t_{{\mathrm{wall}}}}}c^ \ast .$$

To eliminate c^*^, we use that the current across all elements is equal *I*^sleeve^ = *I*^gap^. The cytoplasmic sleeve is an straight annulus of length *t*_wall_ and conductive area *A*_1_, hence the current in the sleeve is

11 $$I^{{\mathrm{sleeve}}} = \frac{{A_1Dc^ \ast }}{{t_{{\mathrm{wall}}}}}.$$

Similarly, the diffusive current can be found by integrating over the inlet gap interface

12 $$I^{{\mathrm{gap}}} = - {\int}_0^{2\pi } r {\mathrm{d}}\theta {\int}_0^h {\mathrm{d}} z\left( {D\partial _rc} \right) = \frac{{2\pi Dh}}{{\ln (R/a_{\mathrm{o}})}}(\Delta c - c^ \ast ).$$

The gap height *h* is pressure-dependent (Eq. (1)), and interactions with the channel walls should be included in the analysis of the diffusion process as *h* approaches the size *s* of the solute molecule. This reduces the diffusion coefficient *D* in Eq. (12) by the factor *D* → *DH*(*λ*), where

13 $$\begin{array}{c}H(\lambda ) = 1 + \frac{9}{{16}}\lambda \ln \lambda - 1.19358\lambda \\ + \, 0.4285\lambda ^3 - 0.3192\lambda ^4 + 0.08428\lambda ^5,\end{array}$$

and *λ*(Δ*p*) = *s*(*h*(Δ*p*))^−1^ is the relative solute size^52^. Accordingly, the gap current becomes

14 $$I^{{\mathrm{gap}}} = \frac{{2\pi DH(\lambda )h}}{{\ln (R/a_{\mathrm{o}})}}(\Delta c - c^ \ast ).$$

**Fig. 7:**
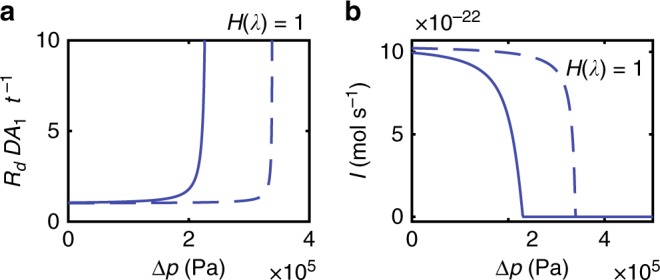
The impact of pressure on PD resistance and transport. **a** Normalized diffusion resistance *R*_d_*DA*_1_*t*^−1^ (Solid line) plotted as a function of turgor pressure difference Δ*p*. **b** Diffusion current Δ*c*(*R*_d_)^−1^ plotted as a function of turgor pressure difference Δ*p* for a concentration difference Δ*c* = 10 μM (Solid line). The transport currents decline in sync with increasing resistance. Dashed lines show results without steric interactions, i.e. *H*(*λ*) = 1. We use *s* = 0.9 nm for the Stokes diameter of common fluorescent dyes^53^

Eliminating c^*^, we can finally express the current as

15 $$I = \frac{{\Delta c}}{{R_{\mathrm{d}}}}$$

where the diffusive resistance *R*_d_ is

16 $$R_{\mathrm{d}}(\Delta p) = \frac{{t_{{\mathrm{wall}}}}}{{DA_1}}\left[ {1 + \frac{{A_1}}{{t_{{\mathrm{wall}}}}}\frac{{\ln (R/a_{\mathrm{o}})}}{{2\pi h(\Delta p)}}\frac{1}{{H(\lambda )}}} \right],$$

as illustrated in Fig. 7

.

## Data Availability

The source data that support the findings of this study and the code used for analysis are available from the corresponding author upon request.

## Source data

Source Data
